# An analytical model to quantify the impact of the propagation of uncertainty in knee joint angle computation

**DOI:** 10.1080/23335432.2022.2108898

**Published:** 2022-08-18

**Authors:** Mickael Fonseca, Stéphane Armand, Raphaël Dumas

**Affiliations:** a Univ Eiffel, Univ Lyon 1, Lbmc Umr_t 9406, Lyon, France; b Kinesiology Laboratory, Geneva University Hospitals and University of Geneva, Geneva, Switzerland

**Keywords:** Joint coordinate system, attitude vector, Euler and cardan angles, knee kinematics, cross-talk, reproducibility

## Abstract

Joint kinematics are typically described using Cardan angles or the attitude vector and its projection on the joint axes. Whichever the notation used, the uncertainties present in gait measurements affect the computed kinematics, especially for the knee joint. One notation – the attitude vector – enables the derivation of an analytical model of the propagation of uncertainty. Thus, the objective of this study was to derive this analytical model and assess the propagation of uncertainty in knee joint angle computation. Multi-session gait data acquired from one asymptomatic adult participant was used as reference data (experimental mean curve and standard deviations). Findings showed that an input uncertainty of 5° in the attitude vector and joint axes parameters matched experimental standard deviations. Taking each uncertainty independently, the *cross-talk* effect could result from uncertainty in the orientation of either the attitude vector (intrinsic variability) or the first joint axis (extrinsic variability). We concluded that the model successfully estimated the propagation of input uncertainties on joint angles and enabled an investigation of how that propagation occurred. The analytical model could be used to *a priori* estimate the standard deviations of experimental kinematics curves based on expected intrinsic and extrinsic uncertainties.

## Introduction

Reproducibility studies have been performed in the literature to evaluate different sources of variability in gait analysis (McGinley et al. 2009; Wren et al. 2011). Some studies have performed a sensitivity analysis on joint axes for a given type of joint motion (Della Croce et al. 1999; Fonseca et al. 2020). More specifically, knee joint kinematics is known to be prone to non-linear error propagation, which results in the well-known kinematic effect of *cross-talk* (Baudet et al. 2014; Pothrat et al. 2015). Cross-talk occurs when the joint axes do not correctly agree with the joint motion for its degrees of freedom. For instance, if the defined flexion-extension axis of the knee is not correctly defined, the varus-valgus and rotation axes are impacted and part of the flexion-extension motion will be translated to those axes.

Joint angles are computed by evaluating the continuous movement of one segment with respect to its adjacent segment. This motion has typically been expressed using two mathematical methods: the Cardan sequence of rotations (Chao 1980; Wu and Cavanagh 1995) and the attitude vector, also commonly referred to as the helical axis or screw axis (Woltring 1991). The Cardan sequence of rotations represents overall joint movement during a set of three rotations about three joint axes: one embedded in the proximal segment (**e**_1_), one *floating* (mutually orthogonal to the two others, **e**_2_) and one embedded in the distal segment with respect to the joint (**e**_3_). These three axes are referred to as the joint coordinate system (JCS, which is a non-orthogonal coordinate system). Due to its easy interpretability, the Cardan sequence of rotations has been recommended as the most adequate for measuring angles in gait analysis (Wu and Cavanagh 1995; Wu 2002). This recommendation was recently extended to the interpretation of joint (*i.e*. intersegmental) moments (Derrick et al. 2020). On the other hand, the attitude vector describes the joint movement by a single axis and consequently it is not affected by the cross-talk phenomena. Comparisons of joint (Cardan) angles and attitude vectors projected (in a non-orthogonal way) on the three joint axes have demonstrated some differences in the kinematic curves and different sensitivities to experimental errors (Ramakrishnan and Kadaba 1991; Woltring 1994; Chéze 2000; Rouhani et al. 2012).

The measurement of gait data, as any other measure, is prone to measurement error. Thus, any complete acquired measurement is accompanied by a quantitative level of measurement uncertainty. Uncertainty is a parameter associated with any measurement that characterizes a dispersion of values around the true value measured (Farrance and Frenkel 2012). In terms of the propagation of uncertainty, the variability in kinematic curves can be understood to depend on the intrinsic variability of joint motion and on the extrinsic variability of the definition of the three joint axes. The intrinsic variability is associated with the ability of a subject to perform repeated movements, and it is considered as an irreductible variability. On the other hand, extrinsic variability is related to the error of measurement and it is characterized by a combination of factors (e.g. placement of the reflective markers, soft tissue artifacts, calibration of motion capture system). To the best of our knowledge, no previous attempts have been made to separate the intrinsic and extrinsic variabilities in the measurement of knee joint kinematics. Intrinsic variability is linked to the movement of the joint itself, independently of any coordinate system, and it can be assessed by looking at the dispersion of the knee’s rotation angle *θ* and of the orientation of the rotation axis **k**. In other words, intrinsic variability is dependent on the ability of the subject to perform a repetitive movement during gait. Intrinsic variability may be affected by the presence of motor disorders, so it is considered an indicator of gait deviations (Chau et al. 2005). Extrinsic variability arises from the inaccurate measure of the real movement of the subject (whether due to instrumentation, mathematical or human factors), which results in dispersion in the orientation of the joint axes **e**_1_, **e**_2_ and **e**_3_. In other words, it is characterised by the error in the definition of the three axes used to interpret the movement of the joint. The theoretical propagation of uncertainty in joint angle computation can be analysed based on the equations used to project the attitude vector onto the three joint axes. These equations only include dot and cross products, which enable the use of the additive rules for calculating uncertainty components through functional relationships (Farrance and Frenkel 2012).

The objective of this study was to define an analytical model to evaluate the propagation of uncertainty in knee joint angle computation and to investigate the origins of the *cross-talk* commonly observed in knee kinematics. We hypothesised, based on previous findings (McGinley et al. 2009), that input uncertainty of 5° in the rotation angle *θ*, the orientation of the rotation axis **k**, and the orientation of the joint axes **e**_1_, **e**_2_ and **e**_3_ would match the experimental dispersion of knee joint angles. Second, we hypothesised that output uncertainty would be more dependent on extrinsic variability (orientation of joint axes) than on intrinsic variability (rotation angle, orientation of the rotation axis) when propagating each of them independently.

## Methods

### Data Collection

Data to assess typical gait analysis variabilities were collected from a single, healthy, asymptomatic adult male (29.3 years old) weighing 92 kg and 183 cm tall, over five sessions performed within two months by a single examiner. A minimum of eight trials was collected per session. The participant was equipped with 53 markers (14 mm) according to the Conventional Gait Model (Baker 2013) and asked to walk barefoot at a self-selected speed. This marker model was chosen as it is the most used model for full-body kinematics in clinical gait analysis. A 12-camera motion capture system (Oqus7+, Qualisys, Göteborg, Sweden) tracked the marker trajectories at 100 Hz. Gait kinematics was processed using the open-source library PyCGM2, CGM1.1 (Leboeuf et al. 2019).

**Figure 1.**
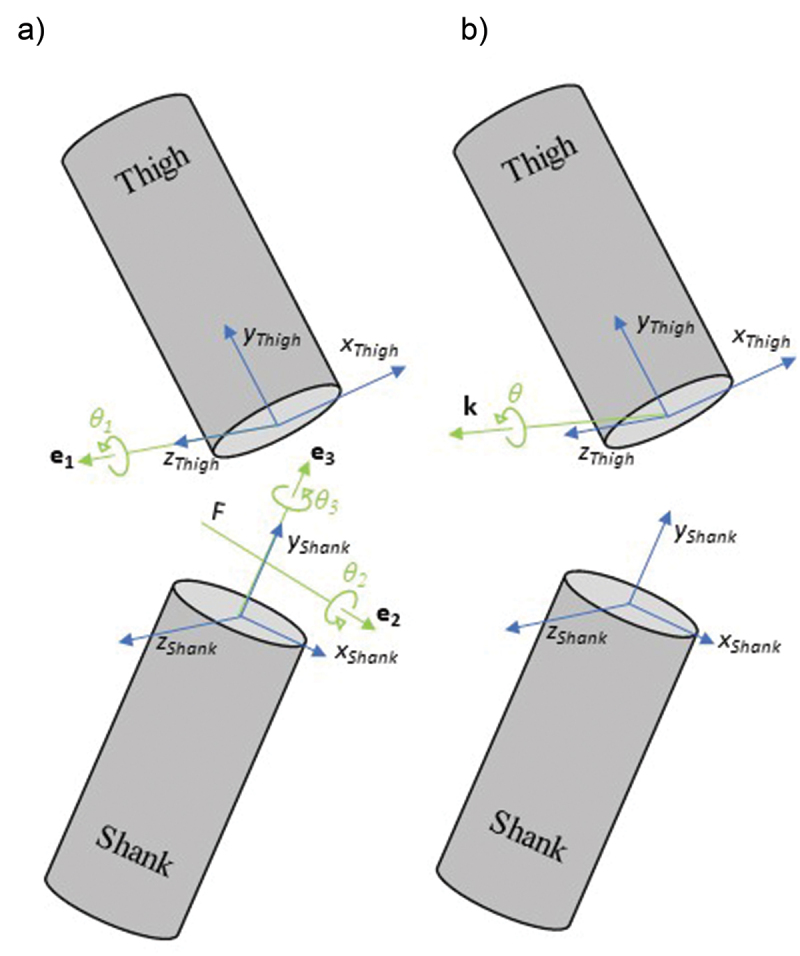
Representation of definition of the cardan angles and axis (A) and attitude vector (B) for the knee joint.

The knee joint represents the motion of the shank segment with respect to the thigh. Both segment’s coordinate systems are defined by a primary axis (Y_Thigh_ and Y_Shank_, superior) between the proximal and distal joint centres. A secondary axis (X_Thigh_ and X_Shank_, anterior) is calculated as the axis orthogonal to a plane defined by the proximal joint center, the wand placed in the segment and the lateral femoral epicondyle or lateral malleolus for the thigh and shank coordinate system, respectively. Finally, the third axis (Z_Thigh_ and Z_Shank_, medial) is defined as the orthogonal axis to the two previously defined (Baker 2013). The definition of the axis and angles for both Cardan angles and attitude vector is represented on Figure 1. Rotation angle *θ* and orientation of the rotation axis **k** were computed using the rotation matrix **R** from the thigh segment to the shank segment. In order to express all quantities in the thigh coordinate system, the definition of the rotation matrix **R** from the thigh segment to the shank segment is considered:

(1) $$\begin{array}{rl} & R=\left[\begin{array}{c} X_{Shank}∙\;X_{Thigh}\;\;\;\;Y_{Shank}∙\;X_{Thigh}\;\;\;Z_{Shank}∙\;X_{Thigh}\\ X_{Shank}∙\;Y_{Thigh}\;\;\;\;\;Y_{Shank}∙\;Y_{Thigh}\;\;\;\;Z_{Shank}∙\;Y_{Thigh}\\ X_{Shank}∙\;Z_{Thigh}\;\;\;\;Y_{Shank}∙\;Z_{Thigh}\;\;\;\;Z_{Shank}∙\;Z_{Thigh} \end{array}\right]\\ & =\left[\begin{array}{c} -s\theta_{2}.s\theta_{3}.s\theta_{1}+c\theta_{3}.c\theta_{1}\;\;\;\;-c\theta_{2}.s\theta_{1}\;\;\;\;s\theta_{2}.c\theta_{3}.s\theta_{1}+s\theta_{3}.c\theta_{1}\\ s\theta_{2}.s\theta_{3}.c\theta_{1}+c\theta_{3}.s\theta_{1}\;\;\;\;\;\;-c\theta_{2}.s\theta_{1}\;\;\;\;-s\theta_{2}.c\theta_{3}.c\theta_{1}+s\theta_{3}.s\theta_{1}\\ \;\;\;\;\;-c\theta_{2}.s\theta_{3}\;\;\;\;\;\;\;\;\;\;\;\;\;\;\;\;\;\;\;\;\;\;\;\;\;s\theta_{2}\;\;\;\;\;\;\;\;\;\;\;\;\;\;\;\;\;\;\;\;\;\;c\theta_{2}.c\theta_{3}\; \end{array}\right]\;\\ & =c\theta .\left[\begin{array}{c} 1\;\;\;0\;\;\;0\\ 0\;\;\;1\;\;\;0\\ 0\;\;\;0\;\;\;1 \end{array}\right]+s\theta \tilde{k}+(1+c\theta ).k^{T}k \end{array}$$

with ‘∙’ the dot product, ‘*c*’ and ‘*s*’ the cosine and sine, ‘~’ the skew matrix notation and ‘^T^’ the transpose vector. As the rotation matrix **R** stands for a ZXY Cardan sequence of rotation (Wu and Cavanagh 1995), the first axis of rotation **e**_1_ is the Z axis for the thigh, that is to say [0 0 1]^T^ expressed in the thigh coordinate system. This rotation matrix is also called direction cosine matrix, meaning that it contains, in column, the components of the X, Y, and Z axes of shank expressed in the thigh coordinate system. The third axis of rotation **e**_3_ is the Y axis for the shank, second column of the rotation (direction cosine) matrix **R**. The second axis of rotation **e**_2_ is the cross product of the two other vectors all expressed in the thigh coordinate system. Attitude vector **k***θ* is computed for the rotation matrix **R** to be further projected onto the joint axes. Cardan angles (for the comparison of experimental variabilities) were also computed from the same rotation matrix **R**. Therefore, from the knowlegde of the X,Y,Z thigh and shank axes in each gait trials, root-mean-square deviation (RMSD) for all the quantities was calculated (equation 1) to assess inter-session and intra-session variabilities. Furthermore, the mean values for the rotation angle *θ* and for components of the rotation and joint axes vectors (**k, e**_2_ and **e**_3_) expressed in the thigh coordinate system were also computed for all the gait trials.

The mean duration of the stance phase was calculated from all the trials. It was 61.6% of the gait cycle, with the remaining percentage of the gait cycle referent to the swing phase.

### Analytical model of the propagation of uncertainty

Equations (2–4) define the non-orthogonal projection of the attitude vector (rotation angle *θ* and rotation axis **k**) onto the joint axes (flexion–extension **e**_1_, adduction–abduction **e**_2_ and internal–external rotation **e**_3_) (Desroches et al. 2010). These three non-orthogonal axes are called joint coordinate system (Wu and Cavanagh 1995; Wu 2002). The symbols ‘x’ and ‘’. designate cross products and dot products, respectively.

(2) $$\theta_{1}=\;\frac{\left(e_{2}\times e_{3}\right)\cdot k}{\left(e_{1}\times e_{2}\right)\cdot e_{3}}\theta$$

(3) $$\theta_{2}=\;\frac{\left(e_{3}\times e_{1}\right)\cdot k}{\left(e_{1}\times e_{2}\right)\cdot e_{3}}\theta$$

(4) $$\theta_{3}=\;\frac{\left(e_{1}\times e_{2}\right)\cdot k}{\left(e_{1}\times e_{2}\right)\cdot e_{3}}\theta$$

To apply the rules for calculating uncertainty components (Farrance and Frenkel 2012), independent variables must be considered. First, the second joint axis (**e**_2_) is defined as the mutually orthogonal vector to the two others, as described in equation (5).

(5) $$e_{2}=\frac{e_{3}\times e_{1}}{\left|\left|e_{3}\times e_{1}\right|\right|}$$

Then, the fact that the different axes are normalised vectors is taken into account by considering two uncertain components only and computing the last one (corresponding to the main direction), as in Equations (6–8).

(6) $$k_{z}=\sqrt{1-{k_{x}}^{2}-{k_{y}}^{2}}$$

(7) $$e_{1z}=\sqrt{1-{e_{1x}}^{2}-{e_{1y}}^{2}}$$

(8) $$e_{3y}=\sqrt{1-{e_{3x}}^{2}-{e_{3z}}^{2}}$$

Therefore, the uncertainty parameters used as inputs for Equation (8) are *θ, k_x_,k_z_,e_1x_,e_1y_,e_3x_* and *e_3z_*.

Equation (9) was adapted from the published rules from the evaluation of standard uncertainty of a measurand *y* through the functional relationships between uncorrelated measured variables *x* (Farrance and Frenkel 2012). It describes the squared standard uncertainty $u^{2}$ of *y* = {*θ*_1_, *θ*_2_, *θ*_3_} by appropriately combining the squared standard uncertainties in the input quantities *x* = {*θ, k_x_,k_z_,e_1x_,e_1y_,e_3x_* and *e_3z_*}. Input *x* variables are all considered uncorrelated with random uncertainty components. Variables g and f represent the denominator and numerator of the equations defining the non-orthogonal projections of the attitude vector onto the joint axes in Equations (2–4).

(9) $$u^{2}\left(y\right)={\left(1/g\right)}^{4}\left[{\left(\frac{g\partial f}{\partial x_{1}}-\frac{f\partial g}{\partial x_{1}}\right)}^{2}u^{2}\left(x_{1}\right)+{\left(\frac{g\partial f}{\partial x_{2}}-\frac{f\partial g}{\partial x_{2}}\right)}^{2}u^{2}\left(x_{2}\right)\text{\textbackslash{}break}+\ldots +{\left(\frac{g\partial f}{\partial x_{7}}-\frac{f\partial g}{\partial x_{7}}\right)}^{2}u^{2}\left(x_{7}\right)\right]$$

The partial derivatives with respect to $x_{i}\frac{\partial f}{\partial x_{i}}and\frac{\partial g}{\partial x_{i}}$ were computed using the Matlab® (R2016b) symbolic toolbox (The Mathworks, Inc, Massachusetts) and then replaced by the mean values of *x* calculated experimentally and the targeted values of input squared uncertainty $u^{2}\left(x\right)$, for each parameter, to compute the output uncertainties $u^{2}\left(y\right)$. In this final step, the input uncertainties in the axes’ orientations were described as a cone of solid angle *α*, as in Equation 10, as an example for rotation axis **k**.

(10) $$k_{x}=k_{y}=\;tan(a_{k})$$

### Testing Procedure

In order to test the first hypothesis, the input uncertainties (*u*(*θ*), *u*(*α_k_*), *u*(*α_e1_*), *u*(*α_e3_*)) were set to 2°, 5° and 10°. As the objective is to evaluate how close a 5° input uncertainty match the experimentally calculated uncertainty between gait sessions, the other included input uncertainties serve for comparison purpose. While 2° has been reported previously as the threshold for optimal variability in the general gait community (McGinley et al. 2009), the 10° uncertainty serve as a reference for high and unacceptable value for extrinsic variability. The output uncertainties (*u*(*θ*_1_), *u*(*θ*_2_), *u*(*θ*_3_)) estimated using the analytical model of the propagation of uncertainty were compared to the experimental inter-session and intra-session variabilities. The best match discovered among those three input parameters was determined as the value that closely match the variability calculated experimentally between the gait sessions. It was then designated as the reference value to be evaluated in the second test, in which each input uncertainty was propagated independently to test the second hypothesis. The impact of each input uncertainty was analysed from a qualitative point of view to determine which joint angles were affected (i.e. overestimated or underestimated) during which phase of the gait cycle.

## Results

Table 1 represents the RMSD for the experimental variabilities calculated from inter-session and intra-session data considering either rotation angles and Cardan angles of rotation or the attitude vector projected onto the joint axis of rotation. The projection of the attitude vector onto the three joint axes resulted in a variability slightly lower than the variability of the respective joint angles for the flexion–extension *θ*_1_ and internal–external rotation *θ*_3_. Contrarily, the variability in the adduction–abduction angle *θ*_2_ was observed to be comparatively lower than its respective attitude vector projection. The rotation axis orientation was the most variable parameter observed (6.35° relative to inter-session measurements). Overall, inter-session kinematic data were found to be more variable than intra-session data, with means (standard deviation) of 4.25° (1.29°) vs 1.78° (0.76°), respectively.

**Table 1.** Experimental variabilities of extracted rotational parameters for the knee joint during gait cycle experiment measurements. RMSD for within sessions (intra-session) and between sessions (inter-session).

	RMSD (in °)	
	Inter-session	Intra-session
Rotation angle	3.12	1.99
Orientation of the rotation axis	6.35	3.01
Flexion–extension angle (Cardan)	5.06	2.36
Projected attitude vector onto **e**_1_	5.05	2.34
Adduction–abduction angle (Cardan)	2.21	0.59
Projected attitude vector onto **e_2_**	2.87	0.85
Internal–external rotation angle (Cardan)	4.77	1.58
Projected attitude vector onto **e_3_**	4.58	1.50

**Figure 2.**
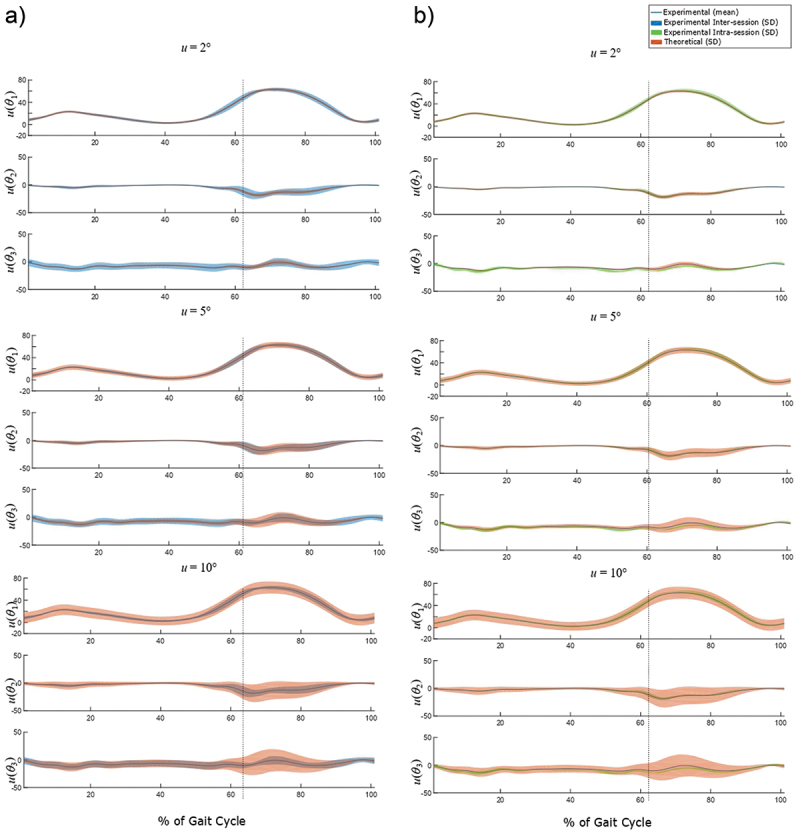
Comparison between the experimental variabilities and the theoretical standard uncertainties (*u*) corresponding to 2°, 5° and 10° of input uncertainty in rotation angle *θ*, the orientation of the rotation axis **k** and in the orientation of joint axes **e**_1_ and **e**_3_. The dotted blue line separates the stance and swing phases. The presented output uncertainties represent the movement of flexion-extension (*u(θ_1_))*, varus-valgus (*u(θ_2_))* and internal-external rotation (*u(θ_3_)).*

Figure 2 compares the experimental variabilities and estimated theoretical uncertainties, using the 2°, 5° and 10° input values. Except for *θ*_1_ during the swing phase and *θ*_3_ during the stance phase, where the best matches with experimental variability were obtained with the input uncertainties of 2° and 10°, respectively, results obtained with an input uncertainty of 5° best matched experimental variability.

**Figure 3.**
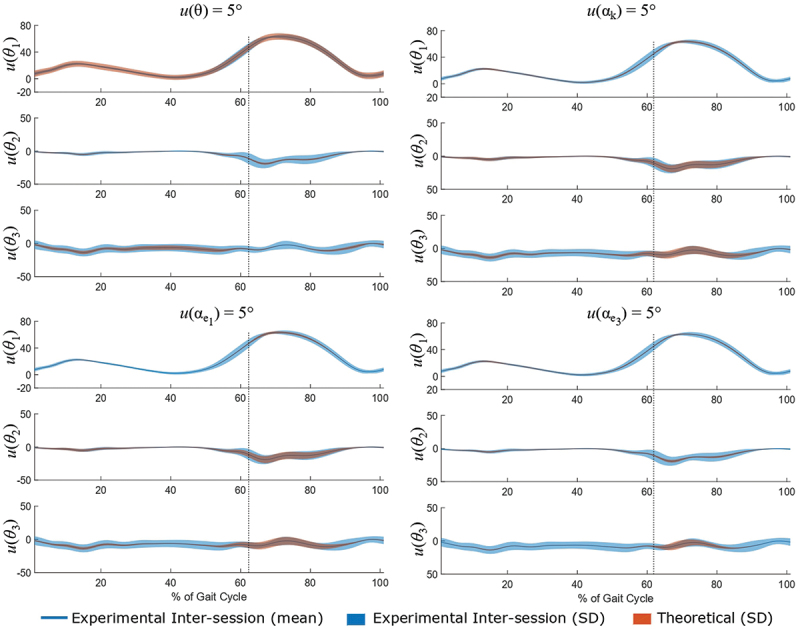
Impact of the standard input uncertainty (*u*) of 5° in the rotation angle *θ*, the in the orientation of the rotation axis **k** and in the orientation of joint axes **e**_1_ and **e**_3_ on knee joint angles. The solid blue line and the blue corridor represent the mean and standard deviation of inter-session experimental variability, respectively, and the red corridor represents the theoretical standard uncertainty (*u*). The dotted blue line separates the stance and swing phases. The presented output uncertainties represent the movement of flexion-extension (*u(θ_1_))*, varus-valgus (*u(θ_2_))* and internal-external rotation (*u(θ_3_)).*

The qualitative analysis of the impact of a 5° input uncertainty in each parameter separately is shown in Table 2. Furthermore, Figure 3 demonstrates the impact of a 5° input uncertainty in each of the rotation angle *θ*, the orientation of the rotation axis **k**, and the orientation of the joint axes **e**_1_ and **e**_3_. The flexion–extension angle *θ*_1_ was the most affected by the uncertainty in *θ*, whereas uncertainty in the other three parameters resulted in very low variability compared to the experimental variability. Moreover, the uncertainty in *θ* resulted in an overestimation of the variability of most of the stance phase (approximately 0%–55%), initial swing (approximately 65%–72% of the gait cycle) and terminal swing (approximately 95%–100% of the gait cycle). For the remaining sub-phases of the gait cycle (55%–65% and 72%–95%), the theoretical corridor matched the experimental corridor relatively well. The adduction–abduction angle *θ*_2_ closely matched the corridors for uncertainty in the orientation of **e**_1_ and **k**, except for the initial swing, where uncertainty in both parameters underestimated experimental variability. On the other hand, the uncertainty in *θ* and the orientation of **e**_3_ showed a general underestimation of the experimental variability of adduction–abduction angle *θ*_2_, with a higher difference on the corridors of swing phase. Finally, uncertainty in *θ* resulted in a noteworthy underestimation of experimental variability by half during the stance phase. Uncertainty in the orientation of **e**_3_, however, had almost no impact, and uncertainty in the orientation **e**_1_ and **k** showed an underestimation of experimental variability by approximately a quarter. For the internal–external rotation angle *θ*_3_, on the initial swing, the uncertainty in *θ* resulted in an almost inexistent corridor, the uncertainty in the orientation of **e**_3_ matched well, and the uncertainty in the orientation of **e**_1_ and **k** overestimated the experimental variability. At mid-swing and terminal swing, the uncertainty in all the parameters resulted in very low theoretical variability.

**Table 2.** Qualitative analysis relative to the impact of 5° of uncertainty in each input variable relative to the stance and swing phases. Experimental variability: highly overestimated (++), slightly overestimated (+), good match (0), slightly underestimated (-) and highly underestimated (–).

	*θ_1_*		*θ_2_*		*θ_3_*		
*u = 5°*	Stance	Swing	Stance	Swing	Stance	Swing	
						>61.8%–85%	>85%–100%
*θ*	++	0	-	–	-	–	–
**k**	–	–	0	0	-	0	-
**e**_1_	–	–	0	0	-	0	-
**e**_3_	–	–	0	-	–	0	-

## Discussion

The main objective of this study was to define an analytical model to investigate the propagation of uncertainty in the computation of knee joint kinematics. Joint kinematics was expressed as the projection of the attitude vector onto the three joint axes of the knee because this enabled the application of the rules for calculating the components of uncertainty. Experimentally, intra-session and inter-session variabilities were verified as being comparable between the Cardan angles and these projections (Table 1). A slightly lower variability was found for the projection of the attitude vector onto the three knee joint axes as previously reported in the literature (Ramakrishnan and Kadaba 1991; Woltring 1994; Chéze 2000). Two hypotheses were made in this study.

The first hypothesis assumed that an uncertainty of 5° would closely match the experimental variabilities recorded in a gait analysis. This hypothesis was confirmed. Findings showed that an *a priori* input uncertainty of 5° in all the intrinsic and extrinsic parameters matched the experimental variability observed on the three joint angles (Figure 2). This was in accordance with previous studies reporting on reliability in gait analysis (McGinley et al. 2009). An uncertainty of about 5° appears to be a generally accepted result in the gait analysis community. The knee joint, however, does not behave like a hinge with a fixed axis and the orientation of the knee’s rotation axis **k** also seemed to be in accordance, again, with a 5° variation (van den Bogert et al. 2008). By dividing the analysis into stance and swing phases, we concluded that the match between our hypotheses and our experiment was not perfect everywhere, as the variability estimated experimentally was sometimes underestimated or overestimated. However, this finding led us to compare the different sources of uncertainty and their impacts on joint angles. It is important to note that the output uncertainties are not additive: the combined uncertainty is the square root of the sum of the squares of the individual uncertainties (and is less than the sum of them).

Our second hypothesis suggested that the output uncertainty was more dependent on extrinsic variability (the orientation of joint axes) than on intrinsic variability (rotation angle, orientation of the rotation axis), when each variability was propagated independently. This hypothesis was not confirmed. By analysing the propagation of uncertainty (set at 5°, according to our first hypothesis) independently (Table 2 and Figure 3), we found that the impact of the uncertainty in the rotation angle *θ* was significant on the flexion–extension angle *θ*_1_ and that the impact of the uncertainty in the orientation of the rotation axis **k** was very similar to that in the first joint axis **e**_1_. These similar impacts were greatest on the adduction–abduction angle *θ*_2_ during the swing phase of gait, and this was a perfect illustration of the well-known *cross-talk* effect (Baudet et al. 2014). Cross-talk occurs when medial-lateral axis of the thigh does not match with the knee movement axis. In this case, both intrinsic and extrinsic variabilities play roles and the cross-talk phenomena is observed for high flexion of the knee that occurs during the swing phase of walking. In comparison, the orientation of the inferior–superior axis of the shank (joint axis **e**_3_) had the most limited impact. Moreover, as with the *cross-talk* effect, the impact of input uncertainties was not linear. Although the flexion–extension angle *θ*_1_ only seemed to be affected by the intrinsic uncertainty in the rotation angle *θ*, the two other joint angles (*θ*_2_ and *θ*_3_) were affected by all the parameters, and their impact was amplified by higher values of *θ*, at approximately 16% and 70% of the gait cycle. As the first and third joint axes are not orthogonal, it can be inferred that input uncertainty in any of their orientations affects all three joint angles.

One limitation of this approach is the simplified view that it provides, as the theoretical error is estimated using an input uncertainty that is constant throughout the gait cycle. Second, this study presents a qualitative overview of the propagation of uncertainties (using the terms of overestimation and underestimation without giving further metrics). Assuming the same amount of uncertainty in both intrinsic (*θ* and **k**) and extrinsic (**e**_1_, **e**_2_ and **e**_3_) parameters, as well as constant uncertainty throughout the gait cycle, can only offer a simplified view. Therefore, this was purposely defined as the objective was limited to using a qualitative approach to demonstrate tendencies in the propagation of uncertainty relative to different input parameters. A final limitation was the study’s population, as data came from a single participant who took part in five sessions with the same examiner, who was also responsible for the experimental setup. Nevertheless, the reference data (mean curve and standard deviations) could be considered as typical values for gait analysis. The propagation of uncertainty, which is assessed qualitatively, should therefore be generalisable in gait analysis.

In conclusion, the analytical model presented in this study helped to improve our understanding of the propagation of uncertainty on knee joint kinematics. Evaluating how variability propagates is important if we wish to understand why the calculation of some joint angles is more uncertain than others, for example. In a clinical context, this could be used to present any experimental joint angle curve with the estimated variabilities for given *a priori* levels of intrinsic and extrinsic uncertainty. Setting this level of uncertainty to 5° would seem appropriate. Due to their specific kinematics, this model may be more useful for investigating the propagation of uncertainty on the knee joint angles and, perhaps, the elbow joint angles than on other joint kinematics.

## Data Availability

The data and code developed to support this study are freely available at https://gitlab.unige.ch/KLab/hel-uncertainty-propagation.git.
